# Spraying sorbitol-chelated calcium affected foliar calcium absorption and promoted the yield of peanut (*Arachis hypogaea* L.)

**DOI:** 10.3389/fpls.2022.1075488

**Published:** 2022-11-28

**Authors:** Tengsheng Li, Qianqian Wei, Wei Sun, Huiting Tan, Yuzhao Cui, Chuanhao Han, Huanyang Zhang, Fanhe Zeng, Mingli Huang, Dongyun Yan

**Affiliations:** College of Environmental Science and Engineering, Qingdao University, Qingdao, China

**Keywords:** peanut, sorbitol-chelated calcium, foliar application, yield, calcium absorption and distribution

## Abstract

The prevalent use of foliar calcium fertilizers in peanut production is inorganic, but calcium absorbed from the foliar has poor availability. Sorbitol-chelated calcium is a novel organic foliar calcium fertilizer that has rarely been studied for application in peanut production. To explore whether calcium absorption and peanut yields can be affected by foliar application of sorbitol-chelated calcium, this study conducted two field experiments using Virginia peanut (Huayu-22) in 2020 and 2021. The five spray treatments included: deionized water (CK), sorbitol (Sor), calcium nitrate (CaN), a mixture of sorbitol and calcium nitrate (SN), and sorbitol-chelated calcium (SC). The yield of peanuts treated with sorbitol-chelated calcium was increased by 12.31-16.63%, 10.22-11.83%, 6.31-9.69%, and 4.18-6.99% compared to the CK, Sor, CaN, and SN treatments, respectively. Sorbitol-chelated calcium had the lowest contact angle due to the wetting effect of sorbitol, which promoted calcium absorption by leaves. Sorbitol-chelated calcium improved the leaf calcium concentration by 13.12-19.32% and kernel calcium concentration by 6.49-8.15% compared to the CK treatment. Foliar fertilization increased the calcium concentration of each subcellular fraction of leaves and changed the distribution of calcium in mesophyll cells. This change was directly observed by transmission electron microscopy. Additionally, spraying sorbitol alone obtained similar effects to spraying calcium nitrate alone, indicating that the benefits of sorbitol itself were not negligible. The results of the principal component and correlation analysis showed that the increase in calcium concentrations and the change in calcium distribution improved the pod traits of the peanut, thus affecting the peanut yield. The above results showed that from the perspective of calcium absorption and distribution, sorbitol-chelated calcium is a more effective foliar calcium fortifier for peanuts and effectively improves peanut yields.

## 1 Introduction

Peanut (*Arachis hypogaea* L.) is an important source of vegetable oil that is rich in proteins, essential vitamins, minerals, antioxidants, polyphenols, and flavonoids (Janila et al., 2013). China is the largest peanut-producing country in the world, and its northern region accounts for more than half of the country’s peanut production area (Zhang et al., 2017b).

Calcium (Ca) is an essential nutrient that plays an important role in plant growth and development and acts as a secondary messenger regulating various metabolic processes, including cell division and apoptosis, the establishment of cell polarity, differentiation, and senescence (Kudla et al., 2012; Zhang et al., 2017a). However, calcium cannot be repartitioned through the phloem route from older to younger tissues of the plant (White, 2003); therefore, calcium deficiency issues often occur in peanuts. The application of calcium fertilizers can significantly increase the rate of peanut germination, promote the growth of peanuts, and improve yield and quality (Pathak et al., 2013; Hamza et al., 2021; Yang et al., 2022). However, compared with the application of calcium fertilizer in the soil, foliar spraying is not considered a particularly effective method to avoid calcium deficiency in peanuts (Hartzog and Adams, 1973; Yang et al., 2022). This may be due to the inorganic state of sprayed calcium, which limits its availability.

Sugar alcohols are endogenously produced nutrients in plants that can participate in cellular metabolism as photosynthetic products and serve as carriers to promote the migration of nutrients in plants (Loescher, 1987; Brown et al., 1999). Recent studies have pointed out that the use of sugar alcohol-chelation technology to chelate calcium ions can convert the inorganic state into an organic state, thereby affecting the absorption and transformation of calcium (Li et al., 2021; Niu et al., 2021). Compared with EDTA-chelated calcium and humic acid-chelated calcium, the production and application of sugar alcohol-chelated calcium is ecological and is considered a new approach for achieving sustainable agricultural development (Bai et al., 2020; Li et al., 2020a; Liu et al., 2021).

However, most of the current research crop species on sugar alcohol-chelated calcium are economic crops, such as fruits and vegetables, but they have little focus on field crops such as peanuts (Li et al., 2021). The reported effect of inorganic calcium on peanuts is much higher than that of organic calcium. In addition, researchers have focused more on the effect of metal ions on crops and therefore have often ignored the role of sugar alcohol ligands in affecting plant growth. This creates confusion in understanding the mechanism of the benefit of sugar alcohol-chelated calcium.

Sugar alcohol is a general term for polyols such as sorbitol, mannitol, and erythritol, and our previous research found that the chelation performance of sorbitol and inorganic calcium is better than that of other polyols (He et al., 2019). Therefore, we synthesized sorbitol-chelated calcium with a chelation rate of nearly 100% in the laboratory and evaluated its effect on peanuts for two years. We hypothesized that sorbitol-chelated calcium could be more readily absorbed by peanut plants from the leaf surface, thereby promoting peanut growth and yield.

## 2 Materials and Methods

### 2.1 Preparation and analysis of sorbitol-chelated calcium

Sorbitol-chelated calcium was prepared from sorbitol, calcium nitrate, and deionized water. Specifically, sorbitol, calcium nitrate, and deionized water were mixed in an appropriate molar ratio in a beaker, and then the dissolved raw materials were placed in a magnetic stirrer (DF-1, Jiangsu Zhongda Instrument Technology Co., Ltd, China), and maintained for 35 minutes at 65°C with a speed of 180 r min^-1^. After natural cooling to room temperature, sorbitol-chelated calcium, the novel product, was obtained. Using the method by He et al. (2019), the chelation rate of the sorbitol-chelated calcium prepared in this study was determined. After three replicate determinations, the average chelation rate of sorbitol-chelated calcium was close to 100%. The total calcium concentration of the sorbitol-chelated calcium stock solution was 80 g L^-1^. All the pharmaceuticals used above were purchased from China Sinopharm Group.

### 2.2 Trial site and weather data

**Figure 1:**
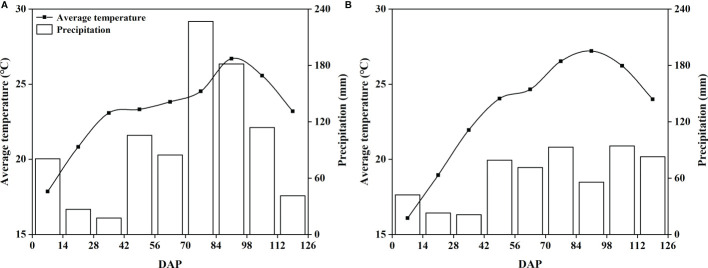
Mean temperature and precipitation at the trial site from 2020 **(A)** and 2021 **(B)**. DAP, day after planting.

The field study was performed from May 2020 to September 2021 in Houhuayuan Village, Qingdao City, Shandong Province, China (36°35’20” N, 120°31’08” E), for two consecutive peanut cultivation seasons (2020 and 2021). The experimental area has a temperate monsoon climate (**Figure 1**). The Huayu-22 peanut cultivar was used, which is a Virginia-type peanut. The soil type belongs to lime concretion black soil in the Chinese Soil Taxonomy (Huang et al., 2017), and the soil texture is clay soil. The terrain is relatively flat, which is very suitable for peanut planting. Before the experiment, one disturbed composite soil sample was collected from 0 to 20 cm soil depth following a five-point sampling method. The chemical properties of the composite topsoil sample were determined according to Bao (2000), and the average values are shown in **Table 1**. The soil nutrient contents were classified as medium.

**Table 1:** Soil properties of the study site.

Year	pH	cation exchange capacity(cmol kg^-1^)	Organic matter (%)	Available N(mg kg^-1^)	Available P(mg kg^-1^)	Available K(mg kg^-1^)	Exchangeable Ca (g kg^-1^)
2020	6.33	25.27	1.32	80.71	29.95	133.41	3.20
2021	6.28	24.69	1.38	88.26	30.84	129.57	3.11

### 2.3 Experimental design and treatments

The field experiment consisted of a total of five treatments composed of (1) deionized water (CK); (2) sorbitol (Sor); (3) calcium nitrate (CaN); (4) a mixture of sorbitol and calcium nitrate (SN); and (5) sorbitol-chelated calcium (SC). The chelation rate of the SN treatment was 0% because sorbitol and calcium nitrate were mixed immediately before spraying to ensure that the components were not chelated. The sorbitol-chelated calcium stock solution was diluted and then used for foliar application. The sorbitol and Ca concentrations used for spraying were 16.36 g L^-1^ and 1.80 g L^-1^, respectively.

The experiment was conducted using a randomized complete block design with four replications. Each plot was 6 m long and 5 m wide (i.e., 5 rows). To minimize the cross-influence, an extra row of guard peanuts was planted surrounding each plot. Sowing dates were on 11 and 3 May in the first and second seasons, respectively. Harvesting dates were 13 and 4 September in the 2020 and 2021 seasons, respectively. The planting density was 180,000 plants per hectare. Compound fertilizer (N: P_2_O_5_: K_2_O = 19:19:19) was applied at a rate of 950 kg ha^-1^ before sowing. The first spray was carried out at approximately 45d (in 2020) or 40 d (in 2021) after planting, and thereafter, foliar fertilization was carried out every two weeks. A total of four and five sprays were applied in 2020 and 2021, respectively. The temperature and humidity of the air in each foliar application were shown in **Table S1**. The dose of the diluted solution was 2.0 L per plot (15 L sorbitol-chelated calcium stock solution per hectare) in the first two applications and 4.0 L per plot (30 L sorbitol-chelated calcium stock solution per hectare) for each of the remaining applications. The foliar application was sprayed at 08:00-10:00 a.m. or 4:00-6:00 p.m. local time on a sunny and windless day. All treatments received identical irrigation, pruning, and control of insects and weeds, which were conducted according to local agronomic practices.

### 2.4 Sample collection and analysis

#### 2.4.1 Yield and pod traits

Peanut maturity was determined according to the hull scrape method (Williams and Drexler, 1981). To avoid border effects, the yield data were collected by randomly selecting twenty plants from the three center rows of each plot at harvest time by hand. The pods were separated from the plants. These pods were dried in the sun and then dried in an electric blast drying oven (101-3AB, Tianjin Test Instrument Co., Ltd. China) at 60°C for 48 h and weighed. The dried pods were hand-shelled. The number of pods containing two seeds (double pods) per plant, the number of mature pods per plant, and the number of total pods per plant were recorded, and the number of immature pods to total pods (pops percentage) was calculated. The pod index (the weight of 100 pods) and seed index (the weight of 100 seeds) were used to represent yield components (Hamza et al., 2021).

#### 2.4.2 Calcium concentrations in peanut leaves, stems and kernels

The leaves and stems were further separated from the plants. These samples were dried in an electric blast drying oven at 105°C for 30 minutes and then dried at 60°C to constant weight. The dried leaves, stems and kernels were ground individually. Then, they were put into a graphite digester (GD-20, Aopule Instrument Co., Ltd., Chengdu, China) for digestion and analyzed by an inductively coupled plasma atomic emission spectrometer (ICP−OES, Avio200, PerkinElmer, Massachusetts, USA) to determine the total concentration of Ca.

#### 2.4.3 Calcium concentrations of leaf subcellular fractions

In 2021, during the flower-pegging stage (~70 DAP), the pod-setting stage (~85 DAP), and the pod-filling stage (~100 DAP), a large number of top third leaves of the main stem of peanut were collected from each plot. The samples were placed in ice boxes, brought back to the laboratory, and quickly rinsed with deionized water and then the veins were removed using a double-sided blade. Cells were separated into three subcellular fractions (cell wall, organelle-containing, and soluble fractions) through differential centrifugation according to the improved method reported by Jiang et al. (2021). Fresh leaf samples for each period were homogenized in cooled extraction buffer with a chilled mortar and a pestle, and the extraction buffer was composed of 250 mmol L^-1^ sucrose, 50 mmol L^-1^ Tris-HCl buffer (pH=7.5), 1.0 mmol L^-1^ dithioerythritol and 5.0 mmol L^-1^ ascorbic acid. The homogenate was centrifuged at 2000 × g for 15 min. The centrifugation was repeated twice, and the sediment was designated as a “cell wall fraction”, which was mainly composed of cell walls and cell wall debris. The supernatant solution was further centrifuged at 10000 × g for 20 min. The resultant deposition and supernatant solution were referred to as the “cell organelle-containing fraction” and the “soluble fraction”, respectively. All of the abovementioned steps were carried out at 4°C. The different fractions were wet-digested with HNO_3_:HClO_4_ at 4:1 (v/v), and the Ca concentrations in the three fractions were analyzed by ICP−OES.

#### 2.4.4 Leaf adhesion and calcium imaging

The contact angles of foliar fertilizers on the peanut leaves were measured by a contact angle meter (Theta, Biolin Scientific, Stockholm, Sweden). After the last spraying in 2021, we collected the top third leaves of the main stem of the peanut from each plot, washed them with deionized water, and wiped them dry. The 2 mm × 2 mm leaf samples near the vein were quickly drawn by a double-sided blade and placed in a precooled syringe containing fixative. The syringe was repeatedly pulled until the leaf samples sank to the bottom. The fixative was prepared from 2% potassium pyroantimonate, 2.5% glutaraldehyde, and 0.05 mmol L^-1^ phosphate buffer (pH 7.5). After fixation for 24 hours, these samples were rinsed with phosphate buffer containing 2% potassium pyroantimonate, fixed again with 1% osmic acid, rinsed with the above phosphate buffer, dehydrated with concentration gradient ethanol, and replaced with anhydrous acetone for dehydration. After embedding, ultrathin sectioning was performed with a Leica microtome (UC6, Leica Microsystems GmbH, Wetzlar, Germany), and the distribution of calcium in mesophyll cells was observed by transmission electron microscopy (TEM, JEM-1200EX, JEOL, Tokyo, Japan). Finally, the black particles deposited in vacuoles, cell walls, or intercellular spaces were analyzed by energy dispersive spectroscopy (EDS) mapping.

### 2.5 Statistical analysis

All data were subjected to a one-way analysis of variance (ANOVA). Data were analyzed using SPSS 25.0 software (IBM SPSS Inc., Chicago, USA). Means were compared using Duncan’s multiple range test at the *P* < 0.05 level. Principal component analysis (PCA) and correlation heatmap drawing were performed on the online tool of the Majorbio Cloud Platform (https://cloud.majorbio.com/page/tools/). Electron microscope images were processed by Photoshop software (Adobe Inc., CA, USA), and the remaining figures were produced by Origin 2018 (OriginLab Inc., Massachusetts, USA).

## 3 Results

### 3.1 Yield, yield components and pod traits

Yield varied significantly across treatments during both years, while the pod index and kernel index changed significantly only in 2021 (**Table 2**). Foliar fertilization was able to increase peanut yield, and in 2020, the yield of the Sor treatment increased significantly compared with that of the CK treatment, while the difference was not significant in 2021. In each year, the SN treatment significantly increased the peanut yield compared with the CK and Sor treatments but did not differ significantly from the CaN treatment. In the two seasons, the SC treatment always produced significantly higher yields than the other treatments. Foliar fertilization reduced the pod index and seed index, but the difference between the five treatments was not significant in 2020. In the second season, the pod index of the CaN treatment and the seed index of the SC treatment were significantly lower than those of the CK treatment.

**Table 2:** Yield and yield components of different treatments in 2020 and 2021.

Treatment	Yield (t ha^-1^)	Pod index (g)	Seed index (g)
2020			
CK	5.51 ± 0.16 d	176.60 ± 1.75 a	83.28 ± 5.00 a
Sor	5.75 ± 0.03 c	168.64 ± 6.90 a	82.03 ± 2.32 a
CaN	5.86 ± 0.04 bc	165.92 ± 3.25 a	81.02 ± 2.75 a
SN	6.01 ± 0.12 b	169.97 ± 2.42 a	81.28 ± 3.39 a
SC	6.43 ± 0.06 a	165.17 ± 2.89 a	79.64 ± 3.93 a
2021			
CK	5.99 ± 0.04 c	206.64 ± 3.51 a	84.83 ± 1.97 a
Sor	6.11 ± 0.11 c	197.48 ± 7.45 ab	83.94 ± 1.35 ab
CaN	6.33 ± 0.07 b	191.31 ± 5.16 b	82.59 ± 1.68 ab
SN	6.46 ± 0.10 b	201.46 ± 7.15 ab	83.11 ± 2.56 ab
SC	6.73 ± 0.07 a	196.71 ± 4.29 ab	81.34 ± 0.43 b

Data are the mean ± standard deviation (SD) (n = 4). Means within each column in each year followed by the same letter were not significantly different based on one-way ANOVAs followed by Duncan’s multiple range tests (P > 0.05). CK, deionized water; Sor, sorbitol; CaN, calcium nitrate; SN, a mixture of sorbitol and calcium nitrate; SC, sorbitol-chelated calcium.

In 2021, the pod traits were significantly affected by foliar application (**Table 3**). For the number of double, mature, and total pods per plant, the SC treatment was always significantly higher than the other treatments, and the CK treatment was always the lowest among all treatments. There was no statistically significant difference in pod traits between the Sor and CaN treatments. The pops percentage in the SN and SC treatments was significantly lower than that in the CK, Sor, and CaN treatments.

**Table 3:** Pod traits of different treatments at harvest in 2021.

Treatment	Double pods number(plant^-1^)	Mature pods number(plant^-1^)	Total pods number(plant^-1^)	Pops percentage(%)
CK	8.37 ± 0.12 c	10.48 ± 0.34 d	18.73 ± 0.06 c	44.07 ± 1.71 a
Sor	8.40 ± 0.24 c	11.87 ± 0.11 c	20.46 ± 0.88 b	43.54 ± 1.02 a
CaN	8.73 ± 0.28 c	11.84 ± 0.14 c	20.42 ± 0.56 b	42.27 ± 1.43 a
SN	9.91 ± 0.41 b	12.92 ± 0.36 b	21.37 ± 0.76 b	38.19 ± 0.73 b
SC	10.75 ± 0.35 a	14.19 ± 0.33 a	23.94 ± 0.77 a	38.90 ± 1.19 b

Data are the mean ± standard deviation (SD) (n = 4). Means within each column followed by the same letter were not significantly different based on one-way ANOVAs followed by Duncan’s multiple range tests (P > 0.05). CK, deionized water; Sor, sorbitol; CaN, calcium nitrate; SN, a mixture of sorbitol and calcium nitrate; SC, sorbitol-chelated calcium.

### 3.2 Contact angles between foliar fertilizers

Contact angle images illustrated the wettability of sorbitol (**Figure S1**). The contact angles of fertilizer droplets of the Sor, SN, and SC treatments with peanut leaves were smaller than those of the CK and CaN treatments, and when sorbitol was combined or chelated with calcium nitrate, the contact angles were the lowest.

### 3.3 Ca concentrations in peanut leaves, stems and kernels

Calcium concentrations in peanuts were significantly affected by foliar spraying (**Table 4**). During the two years, compared with the CK and Sor treatments, the leaf calcium concentrations of the SC treatment were significantly increased but were not significantly different from those of the CaN and SN treatments. In terms of the calcium concentrations of the peanut stem, the SC treatment was statistically and numerically higher than the other treatments, and the difference between the SN and SC treatments in 2020 was significant. In the first season, despite some changes in calcium concentrations, fertilization treatments had little effect on calcium concentrations in kernels. However, the calcium concentrations of kernels treated with CaN, SN, and SC were significantly higher than those of kernels treated with CK and Sor in 2021.

**Table 4:** Ca concentrations (g kg^-1^ DW) of peanut leaves, stems and kernels at harvest in 2020 and 2021.

Treatment	Leaf	Stem	Kernel
2020			
CK	26.49 ± 1.73 c	12.27 ± 0.31 d	0.87 ± 0.04 a
Sor	27.77 ± 1.80 bc	13.61 ± 0.46 c	0.89 ± 0.06 a
CaN	30.06 ± 1.86 ab	13.39 ± 0.39 c	0.90 ± 0.04 a
SN	31.00 ± 2.70 ab	14.60 ± 0.04 b	0.91 ± 0.00 a
SC	31.61 ± 1.27 a	16.78 ± 0.30 a	0.92 ± 0.02 a
2021			
CK	29.81 ± 0.93 c	12.39 ± 0.35 c	0.83 ± 0.05 b
Sor	31.40 ± 1.29 bc	13.04 ± 0.23 bc	0.85 ± 0.01 b
CaN	32.42 ± 0.58 ab	12.91 ± 0.69 bc	0.91 ± 0.04 a
SN	32.78 ± 0.64 ab	13.64 ± 0.20 ab	0.92 ± 0.03 a
SC	33.72 ± 1.38 a	14.13 ± 0.50 a	0.92 ± 0.02 a

Data are the mean ± standard deviation (SD) (n = 4). Means within each column in each year followed by the same letter were not significantly different based on one-way ANOVAs followed by Duncan’s multiple range tests (P > 0.05). DW, dry weight; CK, deionized water; Sor, sorbitol; CaN, calcium nitrate; SN, a mixture of sorbitol and calcium nitrate; SC, sorbitol-chelated calcium.

### 3.4 Subcellular Ca concentrations in peanut leaves

**Figure 2:**
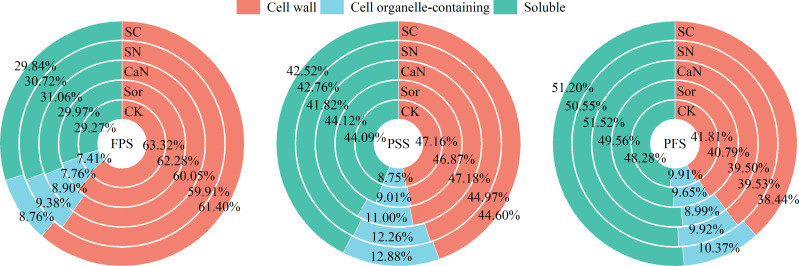
Distribution ratio of subcellular calcium in leaves of peanut at different growth stages. FPS, flower-pegging stage; PSS, pod-setting stage; PFS, pod-filling stage; CK, deionized water; Sor, sorbitol; CaN, calcium nitrate; SN, a mixture of sorbitol and calcium nitrate; SC, sorbitol-chelated calcium.

Foliar fertilization changed the subcellular distribution of calcium in peanut leaves and increased the calcium level of each subcellular fraction (**Table 5**; **Figure 2**). Ca in leaf cells was mainly located in the cell wall fraction or the soluble fraction, and less in the cell organelle-containing fraction (**Table 5**; **Figure 2**). At the flower-pegging stage, the calcium concentrations of each fraction were significantly improved by treatment with sorbitol-chelated calcium (SC) compared with the CK treatment, but the difference was not significant with the CaN or SN treatments. At the pod-setting stage and pod-filling stage, the calcium concentrations of the cell organelle-containing fraction were significantly higher in the SN and SC treatments than in the other treatments, and the differences between the two treatments were significant. Foliar fertilization increased the soluble fraction calcium concentrations, especially in the SC treatment. In terms of total calcium concentration, there was no significant difference between the Sor and CaN treatments, and the difference between the SN and SC treatments was significant at the pod-setting stage.

**Table 5:** Subcellular Ca concentrations (g kg^-1^ FW) in peanut leaves at different growth stages in 2021.

Stage	Treatment	Fractions			Total
		Cell wall	Cell organelle-containing	Soluble	
FPS	CK	2.88 ± 0.01 b	0.34 ± 0.03 b	1.33 ± 0.08 c	4.55 ± 0.09 b
	Sor	2.83 ± 0.08 b	0.35 ± 0.07 ab	1.36 ± 0.08 ab	4.54 ± 0.15 b
	CaN	2.91 ± 0.10 ab	0.43 ± 0.09 ab	1.50 ± 0.13 ab	4.84 ± 0.20 ab
	SN	2.96 ± 0.26 ab	0.46 ± 0.06 a	1.52 ± 0.11 ab	4.93 ± 0.25 a
	SC	3.16 ± 0.15 a	0.45 ± 0.02 a	1.54 ± 0.12 a	5.15 ± 0.25 a
PSS	CK	2.31 ± 0.10 b	0.43 ± 0.03 d	2.16 ± 0.21 b	4.89 ± 0.09 d
	Sor	2.39 ± 0.10 ab	0.46 ± 0.02 d	2.25 ± 0.05 ab	5.10 ± 0.08 c
	CaN	2.48 ± 0.03 ab	0.58 ± 0.04 c	2.19 ± 0.13 b	5.25 ± 0.10 bc
	SN	2.42 ± 0.13 ab	0.66 ± 0.03 b	2.30 ± 0.03 ab	5.37 ± 0.16 b
	SC	2.54 ± 0.12 a	0.73 ± 0.04 a	2.42 ± 0.08 a	5.69 ± 0.12 a
PFS	CK	2.49 ± 0.14 a	0.59 ± 0.01 c	2.88 ± 0.13 c	5.96 ± 0.19 c
	Sor	2.56 ± 0.19 a	0.61 ± 0.02 c	3.11 ± 0.12 b	6.28 ± 0.26 bc
	CaN	2.56 ± 0.10 a	0.58 ± 0.04 c	3.34 ± 0.07 ab	6.48 ± 0.12 ab
	SN	2.59 ± 0.06 a	0.65 ± 0.01 b	3.31 ± 0.16 ab	6.56 ± 0.13 ab
	SC	2.59 ± 0.11 a	0.70 ± 0.03 a	3.45 ± 0.15 a	6.74 ± 0.28 a

Data are the mean ± standard deviation (SD) (n = 4). Means within each column in each stage followed by the same letter were not significantly different based on one-way ANOVAs followed by Duncan’s multiple range tests (P > 0.05). FW, fresh weight; FPS, flower-pegging stage; PSS, pod-setting stage; PFS, pod-filling stage; CK, deionized water; Sor, sorbitol; CaN, calcium nitrate; SN, a mixture of sorbitol and calcium nitrate; SC, sorbitol-chelated calcium.

Foliar application altered the distribution ratio of calcium in subcellular fractions (**Figure 2**). As the growth stage progressed, the proportion of calcium in the cell wall fraction gradually decreased, while the soluble fraction increased continuously. Although foliar fertilization increased the calcium concentrations of each subcellular fraction in mesophyll cells, it reduced the distribution of calcium to the cell wall fraction and promoted the accumulation of calcium in cell organelle-containing and soluble fractions, especially sorbitol-chelated calcium (SC) treatment.

### 3.5 Location of calcium in mesophyll cells using TEM

**Figure 3:**
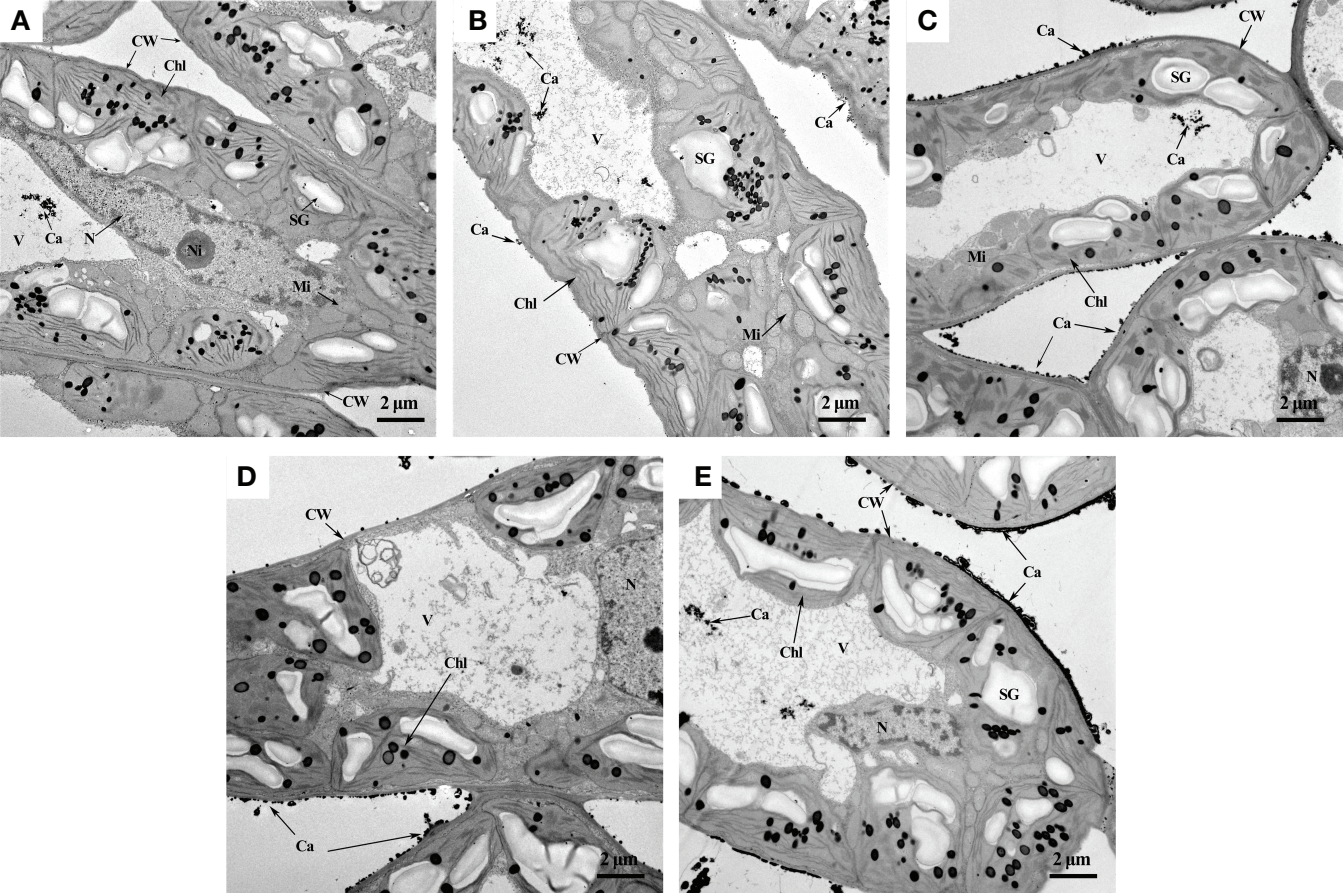
Observation of calcium distribution in mesophyll cells by TEM. **(A)** CK, deionized water; **(B)** Sor, sorbitol; **(C)** CaN, calcium nitrate; **(D)** SN, a mixture of sorbitol and calcium nitrate; **(E)** SC, sorbitol-chelated calcium. CW, cell wall; Chl, chloroplast; V, vacuole; N, nucleus; Ni, nuclear inclusion; SG, starch granule; Mi, mitochondrion; Ca, calcium.

TEM helped us directly observe the changes in calcium distribution in mesophyll cells caused by foliar fertilization (**Figure 3**). The principle is that potassium pyroantimonate can precipitate Ca^2+^ in cells to form calcium pyroantimonate, which cannot be penetrated by electrons, therefore enabling their visualization as dark spots under the electron microscope (Wick and Hepler, 1982). EDS elemental mapping analysis further verified that the black particles distributed in the cell wall and vacuoles were calcium (**Figure S2**). In the CK treatment, more dark spots were observed in the vacuole than in the cell wall, tonoplast, cell membrane, and nuclear membrane (**Figure 3A**). After fertilization treatment, more calcium precipitation was observed in mesophyll cells than in the CK treatment (**Figure 3A**), and the distribution of calcium on the cell wall or intercellular spaces was denser and more uniform in the SC treatment than in the other treatments (**Figure 3E**).

### 3.6 Correlation analysis of yield, pod traits and calcium absorption

**Figure 4:**
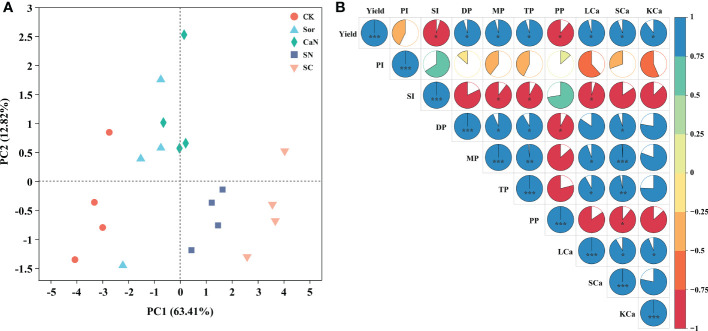
Principal component analysis **(A)** and Heatmap of correlations **(B)** between yield, pod traits and calcium concentration. CK, deionized water; Sor, sorbitol; CaN, calcium nitrate; SN, a mixture of sorbitol and calcium nitrate; SC, sorbitol-chelated calcium. PI, pod index; SI, seed index; DP, the number of double pods per plant; MP, the number of mature pods per plant; TP, the number of total pods; PP, pops percentage; LCa, leaf calcium concentration; SCa, stem calcium concentration; KCa, kernel calcium concentration. *, **, *** represent significance at *P* < 0.05, *P* < 0.01, and *P* < 0.001, respectively.

The results of the principal component analysis (PCA) showed that different treatments were separated, indicating that foliar fertilization significantly affected the yield, pod traits, and calcium concentration of peanuts (**Figure 4A**). The Pearson correlation heatmap further revealed their correlation relationship (**Figure 4B**). Peanut yield was positively correlated with pod number, leaf, stem and kernel calcium concentration. There was a positive correlation between aboveground calcium concentration and pod number. The increase in leaf calcium concentration significantly promoted the increase of kernel calcium concentration. The results of correlation analysis between leaf subcellular calcium concentrations and yield, yield components, and pod traits showed that the calcium concentrations of each subcellular fraction were positively correlated with yield and the number of pods, and negatively correlated with pod index, seed index and pops percentage (**Table 6**).

**Table 6:** Correlation analysis between leaf subcellular calcium concentrations and yield, yield components and pod traits.

Stage	Subcellular	Yield	PI	SI	DP	FP	TP	PP
FPS	Cell wall	0.582^**^	0.163	-0.587^**^	0.419	0.451^*^	0.596^**^	-0.405
	Cell organelle-containing	0.586^**^	-0.244	0.046	0.526^*^	0.510^*^	0.419	-0.557^*^
	Soluble	0.597^**^	-0.252	-0.038	0.523^*^	0.549^*^	0.413	-0.518^*^
PSS	Cell wall	0.535^*^	-0.181	-0.182	0.497^*^	0.542^*^	0.446^*^	-0.256
	Cell organelle-containing	0.880^**^	-0.168	-0.530^*^	0.863^**^	0.873^**^	0.777^**^	-0.797^**^
	Soluble	0.555^*^	-0.191	-0.057	0.495^*^	0.604^**^	0.593^**^	-0.570^**^
PFS	Cell wall	0.319	-0.525^*^	-0.405	0.084	0.181	0.409	-0.013
	Cell organelle-containing	0.679^**^	-0.135	-0.407	0.796^**^	0.756^**^	0.757^**^	-0.599^**^
	Soluble	0.715^**^	-0.399	-0.506^*^	0.520^*^	0.701^**^	0.768^**^	-0.514^*^

The Pearson correlation coefficient was applied in the correlation analysis. FPS, flower-pegging stage; PSS, pod-setting stage; PFS, pod-filling stage; PI, pod index; SI, seed index; DP, the number of double pods per plant; MP, the number of mature pods per plant; TP, the number of total pods per plant; PP, pops percentage. One and two asterisks represent significance at P < 0.05 and P < 0.01, respectively.

## 4 Discussion

### 4.1 Effect of foliar fertilization on peanut yield, yield components and pod traits

Peanut is one of the most important oil crops in the world, and calcium is the most critical factor to realize the high yield of this crop. It is generally believed that foliar application of calcium fertilizer has little effect on increasing peanut yield (Hartzog and Adams, 1973; Yang et al., 2022). However, our study obtained the opposite result. Interestingly, the sorbitol-chelated calcium treatment (SC) was consistently superior to the calcium nitrate (CaN) and a mixture of sorbitol and calcium nitrate (SN) treatments over the two years, and compared with the CK treatment, the sorbitol spray alone (Sor) also promoted an increase in peanut yield (**Table 2**). This is due to (i) calcium in sorbitol-chelated calcium exists in the form of an organic-chelated state, which may reduce the fixation of calcium in phloem and promote the migration and transformation of calcium in plants (Shen et al., 2016); (ii) sorbitol, as a primer of photosynthesis in many Rosaceae plants, has many biological functions in plants, such as energy metabolism, osmotic adjustment and signal transduction (Li et al., 2021). Of course, the penetration and wetting effect of sorbitol reduces the surface tension of liquid spray, which is also an important factor in improving peanut yield. Sorbitol-chelated calcium was found in our study to have a lower contact angle, which means that the fertilizer droplets remain on the leaf surface for a longer time, thereby facilitating the uptake of the fertilizer by the leaves (**Figure S1**). The effect of sorbitol-chelated calcium on improving peanut yield was evident, and the results of this study were also consistent with reports from other crops, including potatoes (Li et al., 2020b), apples (Pei et al., 2018) and tomatoes (Qi et al., 2014). During the growth of peanuts, the interannual variation in rainfall can significantly affect the yield of peanuts, especially in the pod-setting stage (Wang et al., 2021). In our study, between July and August 2020, there was more rainfall than in the same period of 2021 (**Figure 1**), therefore resulting in the occurrence of yield fluctuations that caused lower yields in 2020 than in 2021 (**Table 2**). Foliar fertilization significantly improved pod traits and increased the number of pods per plant (**Table 3**) but seemed to not affect the pod index and seed index (**Table 2**). The results of the heatmap (**Figure 4B**) showed that yield was negatively correlated with the pod index and seed index, but positively correlated with pod numbers, which indicated that the improvement in yield and the decrease in the pod index and seed index in this study were mainly caused by the significant increase in the number of pods per plant.

### 4.2 Effects of foliar fertilization on calcium absorption in peanuts

The level of nutrient content in different organs of peanuts can reflect the nutrient absorption capacity and fertilization has an important impact on the nutrient absorption and distribution of plants. The results of this study showed that the absorbed calcium from peanuts at the time of harvest mainly accumulated in the leaves, followed by stems, and was less distributed in the kernels, which was consistent with previous findings (Zhang et al., 2020). More than 90% of the calcium required for peanut pod development has been reported to be absorbed directly by itself (Mizuno, 1959). Unlike soil fertilization, nutrients sprayed on the leaf surface are mainly absorbed by crops through leaf cuticles and stomata (Niu et al., 2021), but calcium is difficult to redistribute through phloem in plants (White, 2003). However, studies have shown that sorbitol could carry mineral nutrients in the form of chelates (complexes) to migrate in plants, which could improve the efficiency of nutrient transport and alleviate the condition of fruit deficiency (Brown, 1996; Shen et al., 2016). In our study, calcium concentrations in leaves and stems were significantly affected by foliar fertilization, and there was no significant difference in the calcium concentrations of kernels among the CaN, SN, and SC treatments (**Table 4**). When the yield is considered, we still contend that sorbitol-chelated calcium treatment can further enhance the calcium absorption of plants. Sorbitol-chelated calcium droplets had the lowest contact angle (**Figure S1**), which can promote the absorption of Ca by leaves, and then part of the calcium may be transported to pods with sorbitol as a carrier. The higher calcium concentrations in leaves and stems may affect the physiological and biochemical processes and growth of peanuts, therefore indirectly affecting the absorption of calcium from the soil. The correlation results (**Figure 4B**) showed that the high level of calcium concentration in leaves and stems promoted the increase of calcium concentration in kernels. In fact, the absorption of calcium by plants is a complex physiological and biochemical process, and the mechanisms by which the foliar application of exogenous calcium fertilizers affects plant nutrient absorption still require further investigation. The results of PCA and heatmap (**Figure 4**) showed that peanut yield, pod traits, and calcium concentration were significantly affected by foliar fertilization, and there was a positive correlation between them. In other words, foliar fertilization could improve peanut yield by promoting the absorption of calcium by peanuts. However, from the data collected, we could not prove that sorbitol-chelated calcium absorbed by leaves could be redistributed by the phloem and promoted productivity. Therefore, in this study, we believed that the absorption of chelates by peanuts contributes more to productivity than greater phloem mobility.

### 4.3 Effects of foliar fertilization on calcium distribution in peanut leaves

Ca is mainly stored in the intercellular spaces, cell walls, vacuoles, and organelles such as mitochondria and chloroplasts of plant cells (White, 2003; Wang et al., 2004). Among them, the calcium form on the cell wall and intercellular space is mainly calcium pectate, which promotes cell development and maintains the integrity and stability of the cell structure. Calcium oxalate and calcium phosphate accumulated in vacuoles help to regulate ion balance and turgor provision and maintain the stability of cytoplasmic calcium concentration, and soluble calcium plays an important role in maintaining cellular physiological activity (Tan et al., 2017). In our study, foliar fertilization significantly increased the calcium concentration of each fraction of peanut leaves (**Table 5**) and advanced the absorption of Ca. The dissemination of calcium in cells is uneven, and the cell wall and vacuole are the most capacities ranges, but its content in organelles, cytoplasm and the nucleus is relatively low (Wang et al., 2004), which is consistent with our results (**Figure 2**). Studies have pointed out that there are obvious spatiotemporal characteristics of calcium concentrations and their distribution during diverse developmental stages and metabolic processes in plants, such as the anthers of *Impatiens balsamina* (Yang et al., 2020). In the early stage of peanut growth, calcium mainly accumulates within the cell wall (**Figure 2**), which would offer assistance to advance cell division and progress carbon and nitrogen metabolism, subsequently advancing plant development (Hepler, 2005). With the development of the peanut growth stage, the proportion of calcium in the cell wall fraction diminished, but the soluble fraction was the inverse (**Figure 2**). Foliar fertilization could decrease the proportion of calcium in the cell wall fraction, and the advancement effect on cell organelle-containing and soluble fractions was more obvious (**Figure 2**), which demonstrated that the calcium absorbed by leaves could be well utilized by cells or cell organelles, thereby influencing its physiological and metabolic processes. TEM might clearly show the distribution of calcium in mesophyll cells (**Figure 3**), and the Ca distribution in the cell wall and intercellular space treated with sorbitol-chelated calcium was more persistent and uniform. Similarly, Zhou et al. (2018) added CaCl_2_ to mung bean sprouts and observed increased calcium precipitation in the intercellular space and cell wall, enhancing the intracellular accumulation of Ca^2+^. The correlation showed that the increment of calcium concentrations in each fraction was advantageous to make strides in peanut pod traits and increase peanut yield (**Table 6**).

## 5 Conclusion

Sorbitol-chelated calcium is a more effective foliar calcium fortifier than calcium nitrate and a mixture of sorbitol and calcium nitrate. The reasons are as follows: (1) as a chelating ligand, sorbitol could diminish the contact angle between droplets and leaves, and promote the absorption of calcium from foliar fertilizers, thereby increasing the calcium concentrations of peanut kernel. Among them, the 13.12-19.32% leaf calcium concentration and 6.49-8.15% kernel calcium concentration were improved by the sorbitol-chelated calcium treatment compared to the CK treatment. (2) Sorbitol-chelated calcium can significantly increase the calcium concentrations of each fraction of leaves and accelerate the accumulation of calcium in cells. All of these factors together led to the improvement of pod traits and ultimately contributed to yield improvement, which was supported by the results of PCA and correlation analysis. In this study, the yield of peanuts treated with sorbitol-chelated calcium increased by 12.31-16.63% compared with that treated with CK treatment. Overall, from the perspective of calcium absorption and its distribution in mesophyll cells, foliar application of sorbitol-chelated calcium has great potential for promoting peanut growth and development and improving pod yield.

## Data availability statement

The original contributions presented in the study are included in the article/**Supplementary Material**. Further inquiries can be directed to the corresponding author.

## Author contributions

TSL conceived, designed, carried out the experiment and wrote the first draft. DYY provided technical, supervised and complemented the writing. All authors contributed to the article and approved the submitted version.

## Funding

This study was supported by the National Natural Science Foundation of China, China (31972516) and the Key Research and Development Project of Shandong Province, China (2017GNC11116).

## Conflict of interest

The authors declare that the research was conducted in the absence of any commercial or financial relationships that could be construed as a potential conflict of interest.

## Publisher’s note

All claims expressed in this article are solely those of the authors and do not necessarily represent those of their affiliated organizations, or those of the publisher, the editors and the reviewers. Any product that may be evaluated in this article, or claim that may be made by its manufacturer, is not guaranteed or endorsed by the publisher.
