# Supplementary material for: Spraying sorbitol-chelated calcium affected foliar calcium absorption and promoted the yield of peanut (Arachis hypogaea L.)
Source: Front Plant Sci. 2022 Nov 28;13:1075488. doi: 10.3389/fpls.2022.1075488 (PMC9742265; doi:10.3389/fpls.2022.1075488)
Supplement: Supplementary file 1 [file DataSheet_1.docx]

Table S1 Temperature and humidity of the air in each foliar application.

| Year | DAP | Temperature | Humidity |
| --- | --- | --- | --- |
| 2020 | 47d | 25.37 | 68.74 |
|  | 64d | 23.53 | 64.21 |
|  | 76d | 23.30 | 71.69 |
|  | 91d | 26.19 | 83.00 |
| 2021 | 41d | 22.44 | 60.00 |
|  | 53d | 23.12 | 79.76 |
|  | 68d | 27.60 | 51.77 |
|  | 81d | 27.20 | 67.47 |
|  | 96d | 26.55 | 59.43 |

Note: DAP, day after planting.


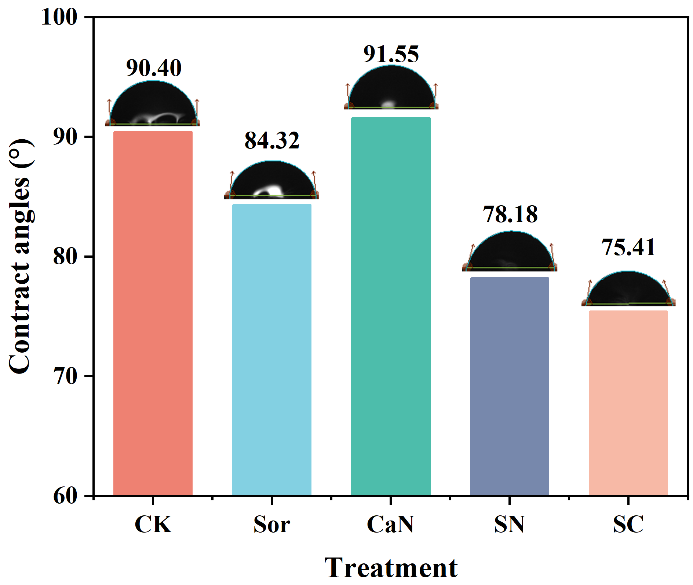


Fig. S1. Contact angles of spray droplets of fertilizer with peanut leaves for five treatments. CK, deionized water; Sor, sorbitol; CaN, calcium nitrate; SN, a mixture of sorbitol and calcium nitrate; SC, sorbitol-chelated calcium.


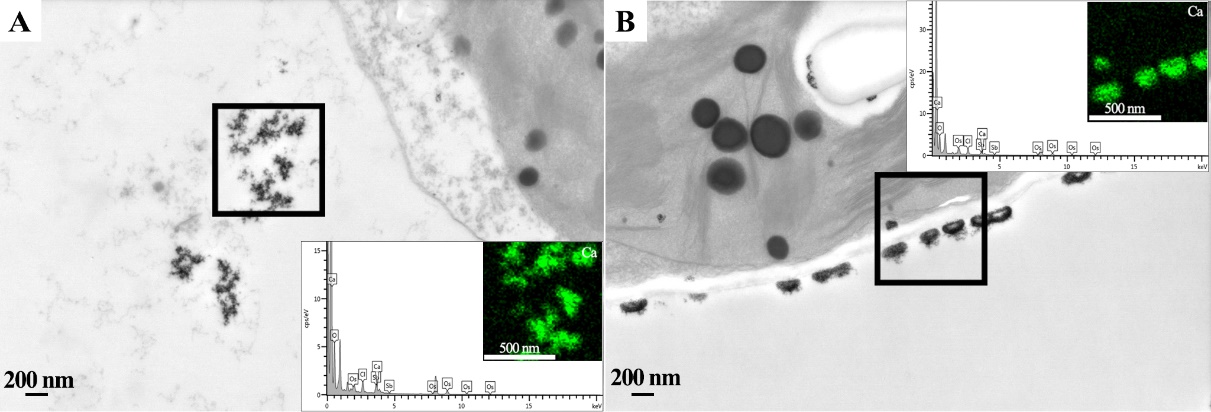


Fig. S2 EDS elemental mapping analysis of black deposits in vacuoles (A) and cell walls or intercellular spaces （B).
